# Next-generation, personalised, model-based critical care medicine: a state-of-the art review of in silico virtual patient models, methods, and cohorts, and how to validation them

**DOI:** 10.1186/s12938-018-0455-y

**Published:** 2018-02-20

**Authors:** J. Geoffrey Chase, Jean-Charles Preiser, Jennifer L. Dickson, Antoine Pironet, Yeong Shiong Chiew, Christopher G. Pretty, Geoffrey M. Shaw, Balazs Benyo, Knut Moeller, Soroush Safaei, Merryn Tawhai, Peter Hunter, Thomas Desaive

**Affiliations:** 1 Department of Mechanical Engineering, Centre for Bio-Engineering, University of Canterbury, Private Bag 4800, Christchurch, New Zealand; 2 Department of Intensive Care, Erasme University of Hospital, 1070 Brussels, Belgium; 3 GIGA In Silico Medicine, University of Liege, 4000 Liege, Belgium; 4 Department of Mechanical Engineering, School of Engineering, Monash University Malaysia, 47500 Selangor, Malaysia; 5 Department of Intensive Care, Christchurch Hospital, Christchurch, New Zealand; 6 Department of Control Engineering and Information Technology, Budapest University of Technology and Economics, Budapest, Hungary; 7 Department of Biomedical Engineering, Institute of Technical Medicine, Furtwangen University, Villingen-Schwenningen, Germany; 8 Auckland Bioengineering Institute, University of Auckland, Auckland, New Zealand

## Abstract

Critical care, like many healthcare areas, is under a dual assault from significantly increasing demographic and economic pressures. Intensive care unit (ICU) patients are highly variable in response to treatment, and increasingly aging populations mean ICUs are under increasing demand and their cohorts are increasingly ill. Equally, patient expectations are growing, while the economic ability to deliver care to all is declining. Better, more productive care is thus the big challenge. One means to that end is personalised care designed to manage the significant inter- and intra-patient variability that makes the ICU patient difficult. Thus, moving from current “*one size fits all*” protocolised care to adaptive, model-based “*one method fits all*” personalised care could deliver the required step change in the quality, and simultaneously the productivity and cost, of care. Computer models of human physiology are a unique tool to personalise care, as they can couple clinical data with mathematical methods to create subject-specific models and virtual patients to design new, personalised and more optimal protocols, as well as to guide care in real-time. They rely on identifying time varying patient-specific parameters in the model that capture inter- and intra-patient variability, the difference between patients and the evolution of patient condition. Properly validated, virtual patients represent the real patients, and can be used in silico to test different protocols or interventions, or in real-time to guide care. Hence, the underlying models and methods create the foundation for next generation care, as well as a tool for safely and rapidly developing personalised treatment protocols over large virtual cohorts using virtual trials. This review examines the models and methods used to create virtual patients. Specifically, it presents the models types and structures used and the data required. It then covers how to validate the resulting virtual patients and trials, and how these virtual trials can help design and optimise clinical trial. Links between these models and higher order, more complex physiome models are also discussed. In each section, it explores the progress reported up to date, especially on core ICU therapies in glycemic, circulatory and mechanical ventilation management, where high cost and frequency of occurrence provide a significant opportunity for model-based methods to have measurable clinical and economic impact. The outcomes are readily generalised to other areas of medical care.

## Background

Intensive care unit (ICU) patients are very difficult to manage safely, effectively and efficiently due to complex and highly variable response to therapy. The cost of intensive care and treatment has risen greatly, primarily due to aging demographics and increasing average life spans (e.g. [1, 2]). In particular, the ability to maintain or improve the equity of access to care is increasingly important and difficult [3–8]. Thus, the major current challenge for ICU care is to improve cost and productivity. In this context, the personalisation of care to better capture the intra- and inter-patient variability in core ICU therapies offers an opportunity to make a significant impact on both the quality and cost of care. In particular, changing from current “*one size fits all*” protocolised care, which can be problematic in the ICU [9–11], to a “*one method fits all*” personalised care, based on physiological models, could provide one significant change to address the demographic tsunami of rising demand and costs.

Computational methods have revolutionised the quality and productivity of output in a wide range of industries over the last two decades, but not nearly so much in medicine [7, 12, 13], where the ability to pay the increasing costs is declining [14]. In ICU medicine, computational physiological models offer a major opportunity to personalise care, combining medical data and model identification methods to generate a “virtual patient” that represents a specific patient in a particular state relative to the system modelled (e.g. metabolic, cardiovascular, pulmonary). In addition, computational methods can draw on an increasing range of physiological models, methods and databases, ranging from simple to detailed and multi-scale models (e.g. [15–47]), which could be used as a foundation for translating computer methods to critical care.

The overall approach relies on the ability to identify patient-specific parameters from data that are both patient-specific and time-varying, and thus manage the intra- and inter-patient variability that defines the typical ICU patient. As a result, such “sensitivities” can be the key to virtual patients and personalised model-based care, as they provide an input–output relationship that reflects patient status and response to the treatment, as well as a metric that can then be used to titrate dose and thus care. Hence, this approach defines the use of deterministic physiological models, and could thus delineate more complex anatomically and biophysically based models (referred to from now on as ‘*physiome models’*) to an informative role, while capitalising upon simpler models for immediate use at the bedside (referred to from here on as ‘*bedside models’*).

In particular, physiome models have the detail and extensive dynamics to provide significant insight into dysfunction at levels that bedside models, with their simpler single organ and/or single system dynamics, cannot [27, 30, 33, 38, 48–58]. Physiome models can also be made patient-specific. However, this task requires significant, often invasive amounts of data, which are precluded by the time and immediacy of the critical care bedside. Finally, while computational tools are advancing and becoming standardised [16, 31, 59–64], the computational intensity and number of variables can preclude direct, immediate use to personalise and guide care, while enhancing the ability to simulate a range of detailed dysfunction. Thus, the physiological scale and/or complexity of physiome models can limit their immediate impact and patient specificity, particularly if the patient-specific condition varies rapidly.

In contrast, the loss of detail in bedside models may hide critical information by lumping it into broader parameters or estimations. There is thus a role for both levels of modeling to integrate and inform each other. Hence, while this review focuses predominantly on the emerging use of bedside, highly patient-specific models and virtual patients, the potential links to physiome models, well reviewed elsewhere [33, 48, 57, 58, 63, 65–68], are also presented.

Bedside models that capture specific fundamental dynamics have long been applied in physiological studies, but are far less used in clinical application, particularly in critical care. However, over the last 10 years, the growing number of model-based sensors or decision support systems in critical care (e.g. [69–90]) has surged, with their successful design, validation and, in some cases, implementation as a standard of care. These results demonstrate the growing interest of using computational models to create personalised solutions to highly variable ICU patients.

Beyond their use in model-based bedside decision support, virtual patients based identified to measurement data are designed to represent the real patients. They can thus be used to design, test and compare different methods of care in silico, whether in real-time or in virtual protocol design. In particular, in silico patient-specific models provide a safe, rapid means to design, prototype and optimise treatment methods. Thus, virtual patients, and their underlying models and methods in particular, provide a tool for both design and implementation of personalised, model-based care.

In addition to safe, rapid protocol design and optimisation, virtual cohorts of virtual patients present the unique opportunity to improve on evidence-based ICU randomised controlled trials (RCTs). Those trials often focus on a single aspect of care or physiology and are thus equally often compromised by lack of generality when clinically applied in general cohorts [91, 92]. The care of any specific dysfunction may be further confounded by a variety of related conditions, such as drug therapy or the body’s own reflex responses. Together, these issues have made designing and implementing effective ICU randomized clinical trials very difficult, making it difficult for the field to move forward as a whole [10, 91–98].

Virtual patients, cohorts and trials offer the potential to advance better solutions taking into account pathophysiological condition as well as intra- and inter-patient variability in response to care. These latter changes in patient state are difficult to control in a RCT as they cannot be predicted [91, 94, 95, 97]. However, they can be easily found retrospectively in clinical data, and thus tested virtually, in silico in designing a clinical trial using a sufficiently broad virtual cohort. Thus, even before implementation one can verify a protocol is robust and safe in the face of inter- and intra-patient variability across a diverse, clinically typical cohort with multiple issues being treated. This capability would, in effect, permit a series of single patient clinical trials to be performed in silico, and to thus optimise a protocol to best manage significant variations in patient behaviour and thus performance.

Hence, a validated in silico virtual trial platform, with associated proper virtual cohorts, would enable the elimination, or a reduction in the number, of phase II/III human trials. For regulatory submissions this potential to replace or augment human trials as an additional, accepted form of evidence is already partly realised [99, 100]. This use of virtual platforms to perform virtual trials shows the complementarity and efficiency gains possible using both in silico and clinical trials to achieve a better or best outcome in a far more efficient manner with less patient burden.

Equally importantly, since virtual trials and patients can readily include device dynamics or simulated clinical errors there is the further opportunity to better link device design and their clinical utilisation in care into both protocol and/or device design (e.g. [25, 101–105]). This point was well made in the case analysis by Viceconti et al. [100], reviewing the literature in this area, and showing two successful examples using virtual patients to develop new products. Thus, there is a critical role in product development for these types of models and methods.

Finally, and in particular, in a joint effort to develop novel devices, clinicians and medical device companies could also use them to develop optimised protocols using existing or new devices designed to fit into clinical workflow with minimal effort. This aspect is especially important wherever human factors at the interface of care and technology play a major role in protocol ease of use. The resulting improved compliance and clinical success, could significantly improve patient outcomes [106–111], by incorporating virtual patients and trials analyses.

This review examines the computational methods and modeling necessary to design virtual patients. Especially, the models and data required are reviewed, and the different levels of validation possible for the resulting virtual patients are discussed. It also notes how virtual trials could be used to design not only protocols, but also to design and test clinical trials before implementation to try and ensure more robust results from these trials. Each section explores the results to date, with particular focus on core ICU therapies managing metabolic, circulatory and pulmonary function. These three areas capture the desire to manage core ICU therapies around glycemic control, cardiovascular and circulatory management, and mechanical ventilation. In each case, variable management of these complex core therapies can be linked to worsened outcomes, increased patient length of stay and thus increased cost. Finally, before concluding, it addresses some of the future possible uses and implications of this emerging fusion of engineering and medical sciences.

## Model types and requirements

A computational physiological model is a mathematical approximation of the observed physical, chemical and/or biological processes, containing certain assumptions in describing these processes. These models can vary significantly in complexity, based on the desired use, and range from simple-compartment models with few parameters (e.g. [18, 21–26, 28, 34–37, 45, 47]) to complicated multi-dimensional network representations or finite element models for similar systems or the entire body (e.g. [15–17, 19, 20, 27, 29–33, 38, 55, 56, 112, 113]).

A virtual patient model (VPM) should have:

- Physiologically relevance.
- Clinically relevance.
- Identifiable treatment sensitivity.

And must also be:

- Identifiable from the data available at the bedside or clinical situation.

*Physiological relevance* of a VPM requires a fixed structure representing the relevant physiological dynamics. While no model can reflect all observed complexity of real world, to be useful, VPMs must be capable to reproduce at least the measurable physiological dynamics in critical care patients to the resolution and accuracy of the sensor readings [18, 114, 115]. The model input and output parameters should be embedded into the mathematics in the model structure describing known physiological dynamics. This requirement thus excludes black-box or generic models, such as neural networks. More directly, while it also ensures that the model captures fundamental physiology, it also sets a lower limit on model complexity.

*Clinical relevance* emphasises models able to be simulated in ‘clinical real-time’, the time between measurements and/or decisions. Model inputs should be the same as those used clinically in treating dysfunction, and outputs should be clinically measured variables relevant to guide care. Equally, it may use measurements not typically used, but for clinical uptake the advantages gained over typical approaches in terms of cost and workflow have to be justified. Hence, the model can fit directly into clinical care with inputs and outputs typically used to guide care, connected by model dynamics matching known clinical input–output responses of the physiological system, all of which ensures clinical relevance. Overall, the requirement of clinical relevance further delineates the possible model structures in size and complexity, where physiological and clinical relevance also defines an optimal model complexity sufficient to warrant a broad rollout by the clinicians.

*Identifiable treatment sensitivity* is a crucial part in designing a virtual patient, as well as applying a VPM to titrate care. Perfectly, care is guided by a correctly predicted patient output in response to treatment inputs. In particular, for virtual patient models, the model output responses to specified model inputs can be used. Critically, sensitivity is defined as the “rate of change” of the output values in response to changes in the input.

For example, in glycaemic control, model-based assessment of insulin sensitivity relates input changes of insulin and nutrition, to expected glycemic response [36, 37, 41, 44, 116–130], which can be monitored and its level and/or variation assessed relative to condition [88, 124, 128, 131–141]. Similarly, in mechanical ventilation, lung elastance determines how changes in the input “a controlled mechanical ventilation volume (pressure)” transfers to the uncontrolled or independent output pressure (volume) response [78–80, 142–151], where this elastance is directly related to the work of breathing and ventilator driving pressure clinicians seek to reduce in titrating mechanical ventilation [148, 149, 152–156]. Again, similar, but simpler, measures of elastance are increasingly used to titrate care [148, 155–160].

In a similar manner, output measures that reflect clinical goals in cardiovascular management, such as beat to beat stroke volume or stressed blood volume [71, 161–166], are increasingly called for clinically to guide care [167, 168]. However, current clinical measures can lack resolution and/or accuracy as they are typically estimated by surrogate measures, are typically measured only intermittently, and can require frequent recalibration during cardiovascular instability [168–173]. Model-based measures [71, 161] could be used to develop sensitivities for inotrope and/or fluid in a pharmacodynamics framework to better guide care with a high resolution model-based metric, as has been done for sedation-agitation management for example [174–176], but has not yet been addressed in this area.

Overall, these sensitivities provide only a few possible case examples. The only limitation in creating these models and defining similar sensitivities is the required physiological relevance in modelled dynamics, and ensuring the model can be practically and feasibly identified, and thus validated, with available clinical measurements [114, 177–182].

In fact, because sensitivities capture patient condition in their response to care, they can be the clinical key in defining and monitoring the intra- and inter-patient variability that defines ICU patients, and thus to guiding therapy, as seen in [117]. They could thus be regularly obtained using such models as part of clinical practice to help assess patient state and response to care, given the advantage of computational models to use clinically available data and system identification methods to obtain a clinically useful sensitivity. In particular, highly sensitive patients need less treatment input or a smaller dose, and vice versa, thus providing a better metric on which to dynamically titrate patient-specific and personalised care. Hence, a physiologically relevant sensitivity metric modeling the relation of clinical input to patient-specific output response to treament is a crucial element in improving care, regardless of whether models are used to identify this metric. In conclusion, this requirement further constrains the potential model structures and organisation, as the model must include such a sensitivity and it must be mathematically identifiable from the clinically available data [114, 177–184].

*Identifiable from the data available* follows immediately from this requirement and refers to mathematical criteria ensuring that the model parameters can be uniquely identified from the available patient-specific clinical data. Identifiability thus implies, that within a clinically relevant time frame a clinically relevant set of data is obtainable that can be used to identify patient-specific model parameters. More specific to virtual patients, this requirement implies it must be feasible to uniquely derive the sensitivity parameter, and all other relevant parameters, from measured clinical data. Thus, identifiable sensitivities are critical in personalising models based on patient data at any given point in time. Pragmatically, with most metrics measured only intermittently and limited real-time data, only limited parameters can be uniquely identified. This limitation places an upper limit on model complexity, at least for direct, immediate use at the bedside (e.g. [70, 121, 177, 180–182, 185]).

This approach is also a minimal approach to the number of variables identified. Insisting on unique identifiability combined with typically limited or sparse data also helps ensure that a model is not over fit to the data. In particular, over fitting can be a major issue when more than one or a few model variables are identified as there is trade-off between them, even if they are uniquely identifiable resulting in a model that does not accurately capture the dynamics or predict well. There are formalised analysis approaches using information criteria and/or practical identifiability analysis to aid in model selection and avoid over fitting and/or trade-off [177, 184, 186–190].

In contrast, increased model complexity can add clinical relevance and detail. Thus, models incorporating several to multi-million degrees of freedom can offer great insight into metabolic, pulmonary, cardiovascular, and, indeed, whole body systems (e.g. [15–17, 19, 20, 27, 29–33, 38, 49, 56, 65, 67, 68]). However, especially given limited available bedside data, their parameter sets cannot necessarily be identified in real-time for use at the bedside in personalised care or virtual patients (to guide care) at this point. Importantly, this limitation does not stop the accrual of data to create a more patient-specific version of a more complex model, but does limit their immediate bedside use for guiding care, and thus the ability to create virtual patients, as well.

Given a suitable set or sets of parameters reflective of real patients, physiome models could have a role in protocol design in simulating real patient conditions with the required high or multi-scale level of detail [32, 33, 38]. Longer term, as care and data progress, enough data could accrue to create a patient-specific, potentially multi-scale bedside version of the physiome model [57, 191], particularly where there are emerging approaches to adapt model complexity to data availability that would unify this transition [192–194].

Overall, a VPM must contain all clinically relevant physiology and physiological dynamics to adjust treatment. Clinically relevant inputs and outputs including an identifiable sensitivity parameter must be included in the model structure. Over time, the evolution of these sensitivity profiles can quantify the intra-patient variability of the patient’s evolution over time as well as inter-patient variability. These requirements allow one to design VPMs, create personalised protocols in silico, and clinically implement those protocols to personalise care.

**Fig. 1:**
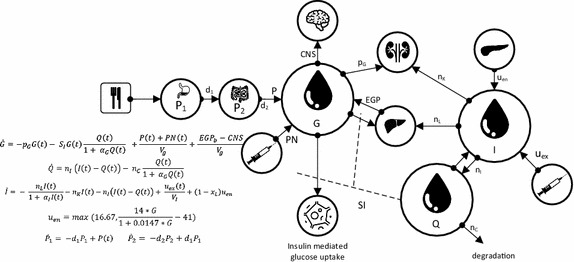
Glucose–insulin pharmacokinetic and pharmacodynamics model used for virtual patients

Figure 1 depicts a metabolic model and its defining equations developed to create virtual patients and design glycaemic protocols [121]. Model inputs include parenteral (*PN(t)*) and enteral (*P(t)*) nutritional carbohydrate uptakes with appropriate appearance dynamics, and the exogenous intravenous insulin input and its kinetics (*u*_*ex*_*(t)*). The VPM structure comprises all relevant nutrition dynamics and insulin kinetics. The pharmacodynamics equation for glycaemia, *G(t),* combines these inputs with the insulin sensitivity, *S*_*I*_*(t),* to deliver a patient-specific glycaemic output response. Depending on data density insulin sensitivity can be identified hourly or more frequently. Patient specific dynamics and their evolution over time can then be reproduced [36, 99, 116, 118, 133, 195, 196].

**Fig. 2:**
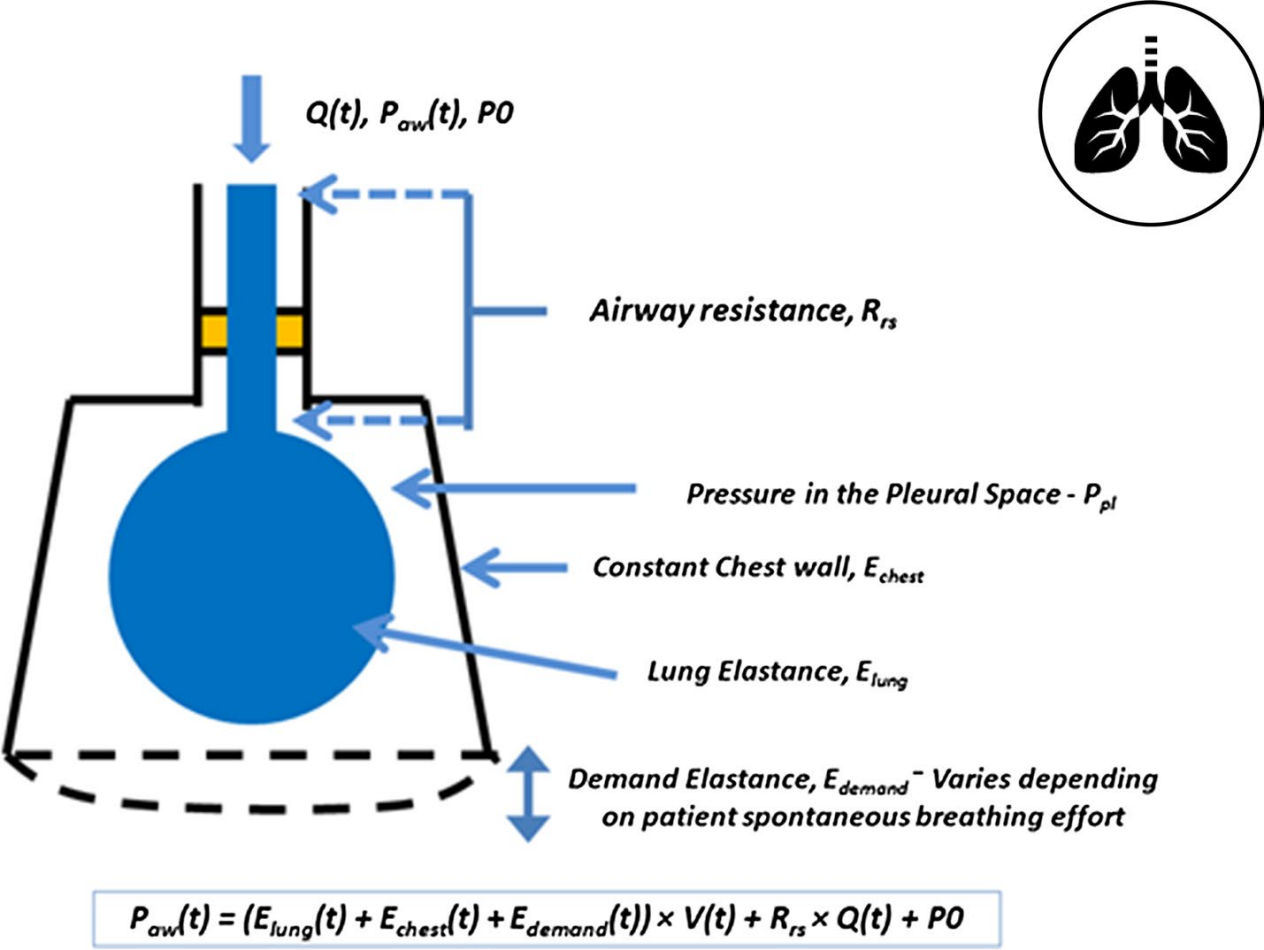
Simple respiratory system ventilation model, where it is modelled as a combination of resistive and elastic components

Similarly, Fig. 2 shows a simple pulmonary mechanics model that can be used to identify breath-specific and patient-specific elastance, the volume (or pressure) response to a controlled pressure (or volume) input in mechanical ventilation. This specific model has been validated on clinical data for both sedated and spontaneous breathing patients [142, 197–200]. As noted, the inputs are volume and flow (or pressure) with concomitant pressure (or volume and flow) independent outputs that are a function of the patient response to ventilation. The model sensitivity determined from these inputs is elastance as the sensitivity, discussed previously, which is used to guide care towards ventilation of patients at, in this specific example, minimal pulmonary elastance.

**Fig. 3:**
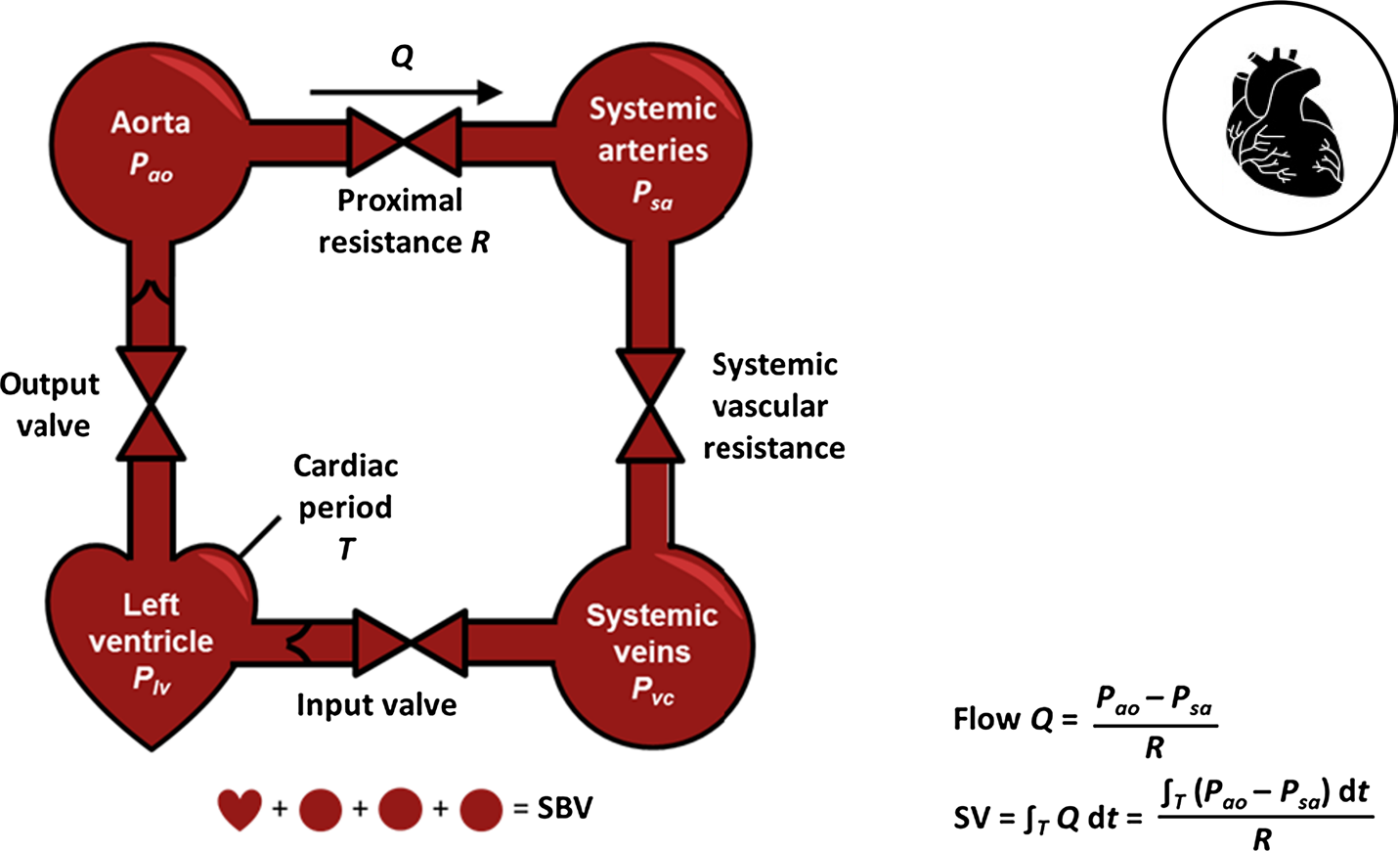
Minimal cardiovascular circuit model used to identify beat-to-beat stroke volume (SV) or/and stressed blood volume (SBV), from which sensitivities to therapy could be developed to help guide care

Last, Fig. 3 shows a specific model and equations for a model-based measure of stroke volume (SV) [161], based on similar prior models [201, 202]. Real-time SV has recently been identified in a consensus statement as a primary variable that would aid the guidance of circulatory management [167]. From this measure, a simple pharmacodynamics models could be created using a standard approach validated in sedation management and other areas, including sensitivities to both inotrope and fluid therapy to aid cardiovascular and circulatory management. It could thus offer a new avenue of addressing key questions in circulatory management around assessing and managing fluid responsiveness and inotrope dosing that are currently major issues in the field (e.g. [167, 203–209]). Similarly, once there is a validated model with a uniquely identified sensitivity, a virtual patient can be created to design and/or implement personalised, model-based care.

When considering physiome models, each might be linked over time to these bedside models. Metabolic behaviour in Fig. 1 would, for example, be better informed by both circulatory and metabolic systems models, particularly in addressing diabetes. Pulmonary mechanics in Fig. 2 are tightly coupled with circulatory dynamics, and, equally, more detailed representations including outcome gas exchange [79, 210, 211] not addressed in just considering elastance and driving pressure, but related to outcome organ function. Finally, circulatory and cardiovascular management using models, like the one in Fig. 3, would be enhanced by more detailed circulatory dynamics in place of lumped parameters. Hence, a hierarchy exists, where this review focuses primarily on the virtual patient and thus the simpler modeling approaches, while noting that the overall idea of reproducible models used at the bedside will readily generalise to other levels and scales of models [57], as data becomes more dense and available.

In summary, the three core areas in this review all have models and/or emerging model-based measures that could be fitted into known model structures. The overall in silico or model-based approach, already in use in glycemic control and metabolic diagnostics, can provide a template for use in other areas, where first pilot and clinical trials are commencing in the pulmonary case [77] and yet to emerge to the best of the authors’ knowledge in the cardiovascular case. Hence, the model definition provided in this section provides a means of evaluating models relative to available clinical data and the clinical application.

## Data required for virtual patients

**Fig. 4:**
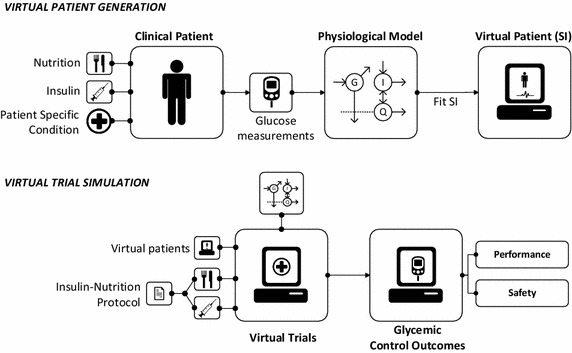
Virtual patient and cohort creation (top) and virtual trials process (bottom) for a metabolic model and system, but generalisable in model and inputs/outputs to any other similar case

As illustrated in Fig. 4, virtual patients are derived directly from VPM and clinical data via system identification of model parameters (“Model types and requirements” section). To identify the desired sensitivities, data is required for all relevant inputs, e.g. for glycaemic control insulin and all forms of nutrition, for mechanical ventilation pressure/volume inputs and positive endexpiratory pressure, and for cardiovascular monitoring the stroke volume and other measures of circulatory pressures and/or flows. Also necessary are measurements of the clinically relevant and modelled output (e.g. glucose, volume/pressure, and stroke volume or other metric, respectively).

A wide range of methods is available to obtain a model-based sensitivity from this input–output data (e.g. [18, 118, 123, 212–222]). Tracked over time, it reveals the evolution of this patient-specific sensitivity to care and patient condition. This trajectory of sensitivity values, captured as a virtual patient, can consequently be employed to calculate how new ways of administering interventions and care (e.g. insulin and/or nutrition) might lead to new, optimized, output results (e.g. blood glucose levels and/or variability), which in turn can be linked to clinical outcomes (e.g. [153, 223–233]).

A further important aspect of VPMs is ensuring relevant physiological and clinically observed dynamics are captured accurately. If the pathophysiological state of patients evolves slowly over time, then these parameters or sensitivities need only be identified at the same pace or slightly faster rate. Similarly, if clinical interventions only vary slowly or their effects can only be captured slowly due to limited or highly invasive measurements, then model parameters should be adapted at a similar rate. There is thus a balance between clinical measurement/intervention rate, and the underlying rate of physical change that can be observed with the data available, and thus identified and validated.

Dysfunction in most ICU patients is typically characterised by fast changing dynamics demanding frequent adjustments of care compounded by, or alongside, significant inter-patient variability in response to care. Thus, the data must be measured with a frequency enabling the identification of these differences in level and rates of evolution in a clinically useful timeframe. However, measurement frequency is subject to a trade-off on clinical workload and quality of care, especially if the data must be manually obtained, entered and/or managed [106, 234–236]. Thus, such an approach also opens the next technological door towards electronic and automated sensing, and eventually, closed loop care systems, as recently emerging with the artificial pancreas in type 1 diabetes [237–249].

Thus, input and measured data must be available at rates appropriate for successfully revealing the observed and model dynamics, which in turn opens new technological opportunities around continuous or automated sensing and data management. If measurements are not frequent enough, the model may miss important changes in patient state and trends to be useful in guiding care. Fortunately, many clinical sensors currently deliver data more frequently than necessary, e.g. cardiovascular pressure catheters providing pressure signals almost continuously, or respiratory pressure and/or flow waveforms are equally sampled at 100–400 samples per second. Further, more and more ICU sensors provide electronic interfaces, allowing automated data access at appropriate rates using standard data acquisition (e.g. pressure signals from a catheter or glucose measurements from a continuous glucose monitor (reviewed for ICU in [250]), significantly reducing the data acquisition workload [234, 251]. Hence, increasingly, it is “only” required to guarantee, that the combination of models and data bring about accurate model-identified estimates of the physiological sensitivities and its evolution.

Overall, it can often be assumed that there is likely to be sufficient and relevant data for all the core areas of care considered here, for which a virtual patient and model-based approach might have impact. Thus, rapidity of data acquisition is not an issue. Equally, it is important to understand the error and/or bias in any sensor to avoid over fitting a parameter, providing an upper limit for the resolution of identified parameters and number of model dynamics required. This upper limit can be informed a priori using existing methods [186, 187], and should also be informed by the underlying knowledge of the physiology. Thus, the size of the model and the ability to identify it remain an issue, but one that increasing data, along with coupling to the bedside scale models discussed in more detail in this review, could be ameliorated in a multi-scale approach [191].

Finally, despite increasing ease to gather data, there is also increasing need for data by researchers. This issue creates the opportunity for data sharing to significantly enhance potential research gains using emerging big data informatics and machine learning, for example. Thus, this review would recommend that groups with large databases consider how data could be safely and ethically shared as is done for example by the ELIXIR database and others in the EU [252, 253].

## Virtual patients, virtual cohorts, and their validation

To this point, the needs and requirements for models, parameter identification methods, and measured clinical data are covered. The metabolic management space is the furthest, having profited from decades of modelling around understanding, diagnosing and controlling diabetes mellitus in outpatients (e.g. [18, 41, 99, 129, 196, 241, 242, 245, 248, 254–257]). Pulmonary modeling for managing mechanical ventilation is emerging towards its first RCT [77], although many models exist (e.g. [28, 78–80, 211, 258]), and cardiovascular and circulatory management is more complex and still emerging. Hence, this section has more focus on the validation of metabolic models and systems, as an example, with broader reference to the needs and requirements applicable to any such model in other areas.

### Virtual patients

Figure 4 shows how virtual patients can be created, where the trajectories of model-based sensitivities offer significant clinical and/or physiological insight independent of any use in the model. It is important to understand virtual patients represent those patient’s specific responses, implicitly encoded by the model structure and parameter values identified from the patient data. If the model structure is appropriate and parameters accurately identified, the outputs will reflect patient-specific condition and evolution. Thus, comparing these trajectories over different conditions offers added insight, e.g. comparing patients with and without a given drug therapy [25]. While, to date, such analyses are limited, the growth of accessible data should enable much more rapid use of model-based sensitivities for research insight and in virtual patients to improve care.

In metabolic systems, insulin sensitivity trajectories have captured the impact of other drug therapies [132, 259], provided insight into the evolution of patient condition over time and thus provided hints how to target better treatment [131, 133, 139], and diagnosed the absence of sepsis [88]. In pulmonary mechanics model-based elastance has been investigated to reveal the impact and effect of recruitment manoeuvres and how they decline over time [152, 260, 261]. The impact of different breathing modes and recruitment manoeuvres [80, 142, 147, 261–263], where clinically assessed, simpler elastance metrics have been insufficient [264]. In the cardiovascular area, model-based methods of determining beat to beat stroke volume or stressed blood volume [71, 161], and other more detailed lumped parameter physiological models used for real-time diagnosis (e.g. [265–268]), have been created with the eventual use to study the effect of inotrope, dosing fluid management with a long term goal of having virtual patients emerge in this area, where further patient-specific guidance would add significant value to a difficult clinical problem [162, 167, 269].

Thus, VPMs and derived virtual patients create an opportunity for defining and optimising new protocols and consequently methods to optimise care. In this way, several protocols for metabolic control have been optimised, at least in part [36, 72, 76, 84, 90, 128, 239, 270–273], and some have gone on to show clinical results very close to those simulated before implementation [90, 274, 275]. Virtual patients can thus be used to enhance protocol development, as well as to create patient-specific protocols using direct modeling and, in some cases, management of the inter- and intra-patient variability in risk-based treatment decisions [72, 84, 85, 89, 119, 120, 128, 270]. Particularly in the glycaemic control, failure to manage patient variability due to the application of fixed protocols has been shown to contribute significantly to the failure of large clinical trials [117]. This evidence is in strong support of the use of virtual patients to develop protocols that directly manage patient variability in ways clinically derived protocols cannot.

Equally, virtual patient models can also be used to analyse existing protocols. Numerous possibilities include testing protocol variations, including timing, sensor errors, errors in dosing, other inputs, and patient variability [36, 101–103, 117, 135, 276], or even the development of robust sensor data management and alarms [277, 278]. In particular, it should be noted that while sensor models are not in the scope of this review, there is a rich literature on validated sensor and device models, which can play a key role in these simulations (e.g. [118, 279–291]). Virtual patient models can also be used to test model assumptions, such as the impact of fixed, unidentifiable physiological model parameter values on performance and/or control when they cannot be identified from the available clinical data [36, 101, 292–295]. Hence, virtual patient models can test model assumptions, the impact of expected or unexpected physiology, the impact of delivery or sensing technologies, or any combination.

Finally, it might be worthwhile to note that all these methods used to create virtual patients have a sound basis over decades of physiological modelling and parameter identification of physiological parameters for research studies. The concept of virtual patients, which to the best of the authors’ knowledge was first introduced for designing a protocol for a standard of care in [272], has significantly extended these early works and can be found in other patient simulators and models (e.g. [85, 99, 102, 123]). In fact, a recent analysis about future ICU research noted its current critical failures, mainly, too often syndromes and specific cohorts are studied that do not generalise and that perhaps in the future “single patient trials” may become more important [91, 93]. Virtual patients capture these single patient trials in silico, and can be used in real-time for decision support in personalised, model-based protocols, uniquely treating each individual patient in a personalised “*one method fits all approach*” or single patient trial.

### Virtual cohorts

Virtual cohorts are simply collections of virtual patients. Since virtual patients can be derived from any group of useful and clinically relevant patient data, virtual cohorts can be designed to be as specific, or, more relevantly, as general as possible. Thus, a virtual cohort can be derived from any such collection of relevant patient data. The data should be as representative and complete as possible of the targeted treatment group or cohort, while ensuring it contains all reasonably expected patient states, patient dynamics and evolution of patient condition, which might occur in practice. Thus, broad and typical, where many randomised trials have very focused and thus potentially atypical cohorts [91].

For example, a glycaemic control virtual cohort with none of the rapid rises or changes in insulin sensitivity that lead to hypoglycaemia and its associated risks in treatment and outcome, would not be representative of observed clinical results to date. The same would hold for the other areas of analysis considered here, including but not limited to sudden desaturation events or the impact of proning, a controversial topic [296, 297], in ventilation on the overall effective respiratory elastance, or the impact of sudden changes in circulatory dynamics. It is the authors’ experience in applying virtual patient models to design protocols and clinical trials, and then implementing them in model-based care, that, in fact, to ensure patient safety and protocol robustness, it is better to slightly over represent outlying cases than to under-represent or miss them.

Consequently, a virtual cohort should cover all clinically relevant patient dynamics observed in care, similarly to the physiological model and clinical data that should represent all relevant patient dynamics observed in care. Failure to comply with this constraint implies a potentially skewed protocol design due to the missing dynamics and thus imposes additional risk to either safety or performance during deployment of a protocol created using this approach. Equally, a skew towards outliers or a certain behaviour that is not representative of the treatment cohort itself can also provide bias in the protocol. This choice may be acceptable as long as all dynamics are represented as any in silico design could ensure robustness across those dynamics in the virtual patient cohort. Thus, these cohorts and episodes should be broad enough in patient numbers and patient diagnosis to capture all clinically observed dynamics, particularly outlying effects or cases. They must also accurately represent what would be found in ICU practice, so that simulations of these virtual cohorts implementing virtual trials yield results that match what is seen, or would be seen, in practice.

The research relevance of this latter point is important as a recent analysis on the state of ICU research noted that clinical trials with limited cohorts often do not generalise to broad cohorts and typical care situations [91]. Virtual cohorts enable the design of effective, general protocols for the typically diverse cohorts seen in practice, yielding a model-based and personalised “*one method fits all*” form of care that robustly translates to all or most patients [273, 298]. The resulting protocols thus offer the ability to design and conduct clinical trials with greater confidence in the ability of the results to generalise.

Virtual trials (Fig. 4) give model-based indications of likely safety and performance outcomes for protocols tested on a particular virtual cohort. Virtual trials require either a treatment independent sensitivity, such as insulin sensitivity in the glycaemic control case, or some way of capturing changes in this sensitivity with time and/or treatment interventions. In the case of the pulmonary modelling, a short or longer recruitment manoeuvre that results in improved lung recruitment will change the underlying elastance sensitivity, or volume response to pressure (or vice versa), but can be used in a protocol to identify a current state and select care. Capturing how these changes in virtual patient based virtual trials on a per-patient or cohort basis is the subject of ongoing work in the field.

### Validation: ensuring the model is “Good”

The critical question with any modelling approach is how to verify if the model is valid in capturing the clinically observed relevant dynamics? Fitting a model to data does not guarantee the identified sensitivity parameters and resulting patient-specific model will accurately capture the patient-specific response to care. Due to model complexity, currently the only practical approach to test validity and thus the identified, patient-specific model parameter values is to test if the identified model accurately predicts the outcome in response to a new clinical input. This test clearly shows whether the patient-specific model and parameter values have accurately captured the patient-specific condition. But, how do we best and with least effort validate such virtual patients and models in practice?

**Fig. 5:**
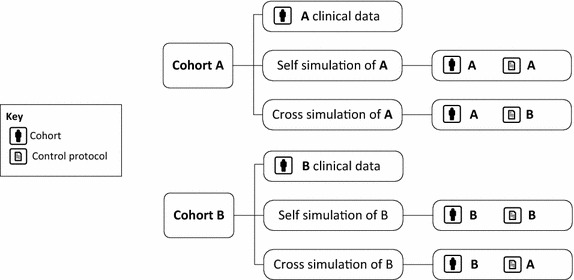
Virtual trial scheme for self- and cross validation example for the two cohorts and protocols

This review proposes three increasingly rigorous validation tests to assess the validity and thus clinical utility of a model and virtual patient cohort:

- *Patient level* A patient-specific model can be identified form a series of clinical data at a given moment in time, from which the outcomes from subsequent clinical inputs can be simulated. Predicted, patient-specific outputs are compared to the measured patient data testing the predictive validity of the virtual patient model. This test assumes an accurate identification resulting in accurate prediction of the patient-specific sensitivity and patient dynamics. However, since patient-specific condition changes over time, within the range of intra-patient variability this test thus validates the patient-specific VPM dynamics.
- *Cohort level before-after* Given a virtual cohort a specific protocol can be simulated and outcomes compared to prior or later application of the protocol in clinical use. Comparing statistical cohort results (median and variability) and individual patient median results assesses if virtual patient model accurately captures cohort dynamics (median and variability) and the central tendency of the individual patients in that cohort. This test validates the models and parameter identification methods over a cohort and specific, individual virtual patients over an entire episode or stay, and thus the overall approach. However, it does not validate specific patients beyond central tendency of their outputs.
- *Cohort level cross validation* This validation test uses data from multiple clinically matched cohorts (typically by severity score, diagnosis or other patient metric) to create matched virtual cohorts. These virtual cohorts can be tested on the original protocol they were given, providing a “self validation” assessing model errors, as above. Cross validation tests the virtual patients of Cohort A on the protocol given to Cohort B with results compared to the clinical results for the (matched) Cohort B, and similarly so for any other cohorts (C, D, etc.). The other cohorts are treated similarly. Figure 5 illustrates the approach. The quality of outcome at the cohort and per-patient levels of each cross validation test can be compared to clinical data from a clinically matched and thus equivalent cohort. These tests assess the independence of the identified patient-specific, model-based sensitivities from the data used to create these virtual patients, including their ability to accurately model the underlying patient dynamics so they provide the expected/same results, as the matched clinical cohort when treated with another protocol.

These three validation tests assess the validity of the model, the identified patient-specific sensitivity parameters, and the ability to accurately design protocols that clinically produce the same results seen in silico at both the patient and cohort levels. The cohort level allows accurate in silico estimation of clinical performance of the protocol when used. The patient level allows accurate evaluation of safety or outlying events, which occur to selected patients in a cohort. Together, these validation tests ensure virtual patient and model validity by ensuring that using them to design a protocol will deliver the same results clinically.

Examples of the first form of validation are found in [121] and have been applied in multiple studies [36, 123, 276]. Importantly, these validation tests do not focus on model fit to data, but the ability to adequately predict a wide range of clinical outcome metrics, or simply, the ability of the model to generalise. Similarly, pilot trials can be used in this form of validation, where targeted clinical data is collected prospectively using a model and protocol (e.g. [89, 299]).

At the patient level, predictions are only as accurate as the ability to capture variation in patient state over the addressed relevant time interval. Assessing the range of error allowable or likely for intra-patient variability can be done, for example, using stochastic models that provide estimates of the range of intra-patient variability [117, 120]. There is also significant work in the area of type 1 diabetes modeling assessing variability of model parameters including physiological and behavioural changes using Bayesian virtual patients and cloning and statistical models [300, 301], which are part of the validation of the Padova type 1 diabetes simulator [99] recently approved for use in some regulatory submissions. However, since many intra-patient variations over a reasonable interval are relatively small, patient level prediction is thus a basic first validation step of the model and sensitivity values, because the ability to capture physiology by predicting outcomes to clinical inputs is a critical first element.

Cohort level before-after validation has only been presented, to the authors’ best knowledge, twice [90, 274]. The main desired goal is to predict cohort level median response and variability (e.g. interquartile range and/or 90% range). This requirement thus wants a model to capture the first and second order cohort level statistics critical to understanding protocol performance and overall risk, and, ideally, also the per-patient central tendency (e.g. median) statistics, as well.

Successful, before-after validations illustrate how in silico protocol design can accurately capture the outcomes of clinical trials, well in advance of the actual clinical use, enabling far faster and safer protocol design. Hence, this form of validation also illustrates the potential of virtual patients in designing safe, effective and personalised new treatment approaches. While such models are now emerging in other areas than glycemic or metabolic control, the requirements noted above could be seen, at this point, as being general across these areas.

Cohort level cross validation has only been demonstrated twice [116, 302], and could be viewed as the most rigorous form of model validation, requiring extensive data and trials in the area even to enable the test. Good results comparing both cohort and per-patient median response in this study validated the underlying model assumptions, showing the identified sensitivity parameter, in this case insulin sensitivity [116], was an accurate representation of patient condition and evolution at a cohort and patient level, independent of the clinical inputs used to identify that value. The second case presented this evidence across three independent ICU cohorts and different clinical practices for two protocols, one model-based and one local clinical protocol [302]. These outcomes thus more conclusively validated these models, methods, and overall virtual trials methodology.

In summary, this section presents three forms of model validation, trying to answer the essential question of “how good are the model and virtual patients created?” The three tests presented are in increasing order of rigor, and provide increasing levels of confidence to a user that the model and virtual trial outcomes will be the same when used in clinical practice. They also require increasing data from pilot or clinical trials, and thus would be obtained as a field matures, where, in this case, the metabolic modeling area is mature and the cardiovascular area is still emerging only now at the first level, for example. However, the overall tests themselves are general and should be applicable to any virtual patient model, method, and eventual virtual trial.

## Further and future possible implications: economics and decision making

This review has presented virtual patients primarily from the viewpoint of their technical potential to solve medical problems in terms of their use to develop, design and clinically apply novel therapeutic approaches. The ability to use their underlying models and identified patient-specific parameters as model-based sensors better assesses patient state and can predict the potential response to therapy. However, these technical and clinical advantages might also flow-on to significantly affect the economics and decision making in health care.

In addition, given the ability to reasonably predict the response to an intervention, whether in real-time or over a virtual cohort, then it would be possible to ascertain the cost of care or required change in care. Any intervention has a cost, including the personnel required to administer it. For glycemic control, these costs may be relatively low for glucose sensing and insulin, although the time cost of care can be high [234]. On the other hand, the cost and clinical impact of catheters in cardiovascular monitoring (e.g. [303–307]) or added cost of each mechanically ventilated patient day [308] can have large impacts on the cost and outcome of care, so the ability to optimise their use and improve outcomes could help alleviate cost pressures. Studies have associated improved levels of glycaemic control with net cost savings [309, 310], at least some of which might be predicted in simulation. Hence, the ability to understand the number of interventions and devices required for a cohort, using virtual trials and locale specific cohorts, could lead to better understanding and/or predicting some of the significant contributors to the cost of care. Overall, as such frameworks emerge, it could become possible to assess, monitor, and track the cost of ICU care in particular settings. A virtual trial framework for protocol design may consider not only for improvements in care and outcome, but also optimisation of resource utilisation in a specific setting.

Finally, as virtual patient methods with the ability to model and predict the response to care improve, it may become possible to determine the impact, or futility, of care. In glycemic control it is already possible to assess a resistance to insulin therapy in some patients. While hyperglycemia is well associated with increased risk of poor outcome and mortality, it does not in any way imply futility for all care of that patient. However, in future, across multiple organ systems and modes of care, it may become more possible to assess overall patient outcomes, creating both the opportunity to avoid prolonging ineffective care, as well as generating significant ethical debate around the legitimate uses and scope of technology in decision making.

## Conclusions and overall summary

The combination of engineering and computer modelling approaches has revealed great potential in intensive care medicine. Easier, more frequent access to data allow increasing ability to utilise computation to enable the model-based design and implementation of better protocols/procedures enabling improved, personalised care. The methods used to design virtual patients and cohorts reviewed in this article are the emerging tip of a much larger iceberg in personalised, and potentially increasingly automated, care in the ICU, and eventually in less acute and out-patient care.

Personalised, patient-specific precision medicine is widely tipped as the next major advance in health care. The transition from “*one size fits all*” protocols with little or no adaptation to manage intra- and inter-patient variability to personalised, time-varying “*one method fits all*” approaches will enable better care and thus improved outcomes compared to current non-computerised care, which lacks the capability to process the available clinical data to achieve these goals. Hence, one immediate and critical key element in achieving these goals is the development and increasing use of robust, accurate virtual patient models and methods addressing major areas of ICU and chronic disease, including the metabolic, pulmonary and cardiovascular systems, as reviewed here.

In particular, the merger of increasingly available clinical data and engineering modelling can create virtual patients and cohorts, which, properly validated, can be used to analyse, design and optimise care, while maximising safety and performance. They also offer the opportunity to test the potential of new devices and drug therapies in silico, far faster and more safely than via a clinical trials based approach, saving time and reducing risk. This review has thus delineated the types and structure of models necessary, including the need for key sensitivity parameters, defined the required and increasingly easily obtained clinical data, and, critically, proposed three levels of model validation to ensure the resulting virtual patients and cohorts are safe, accurate and clinically relevant. The overall result creates a clear outline of the state of the art in each area and their interaction.

In future, virtual patient cohorts might be readily collected and curated from increasingly deployed central electronic patient data management services, for open access use by researchers, industry and/or regulatory bodies. They enable not only in silico testing of new treatments and/or devices, but also the potential for standard in silico testing for regulatory approval. In addition, more rapid design and optimisation of personalised care approaches may well yield significant economic and social benefits from improved productivity in care and patient outcomes.
